# Long-term repetition priming and semantic interference in a lexical-semantic matching task: tapping the links between object names and colors

**DOI:** 10.3389/fpsyg.2014.00644

**Published:** 2014-06-24

**Authors:** Toby J. Lloyd-Jones, Kazuyo Nakabayashi

**Affiliations:** ^1^Wales Institute of Cognitive Neuroscience, Department of Psychology, Swansea UniversitySwansea, UK; ^2^Department of Psychology, University of HullHull, UK

**Keywords:** color, object, name, shape, memory, repetition priming, modality-specific, semantic interference

## Abstract

Using a novel paradigm to engage the long-term mappings between object names and the prototypical colors for objects, we investigated the retrieval of object-color knowledge as indexed by long-term priming (the benefit in performance from a prior encounter with the same or a similar stimulus); a process about which little is known. We examined priming from object naming on a lexical-semantic matching task. In the matching task participants encountered a visually presented object name (Experiment 1) or object shape (Experiment 2) paired with either a color patch or color name. The pairings could either match whereby both were consistent with a familiar object (e.g., *strawberry* and *red*) or mismatch (*strawberry* and *blue*). We used the matching task to probe knowledge about familiar objects and their colors pre-activated during object naming. In particular, we examined whether the retrieval of object-color information was modality-specific and whether this influenced priming. Priming varied with the nature of the retrieval process: object-color priming arose for object names but not object shapes and beneficial effects of priming were observed for color patches whereas inhibitory priming arose with color names. These findings have implications for understanding how object knowledge is retrieved from memory and modified by learning.

## Introduction

Stored knowledge of object color, for instance that a strawberry or stop sign is typically red, can make an important contribution to everyday tasks such as selecting food at the supermarket or using signs when negotiating road traffic. These interactions often require retrieving color knowledge from memory along with other forms of information associated with a particular object or category of objects such as the object name or shape. To understand the properties of the different processing components mediating the memorial retrieval of object-color knowledge however, it is necessary to develop paradigms that tap components selectively. Here, we developed a novel paradigm which engaged the long-term mappings between object names and the prototypical colors for familiar objects and so allowed us to assess effects of learning within the lexical-semantic memory system for object-color knowledge. We examined the role of retrieval processes in the activation of object-color knowledge and the effect they may have on memory performance as indexed by long-term repetition priming (the benefit in performance from a prior encounter with the same or a similar stimulus). In particular, we assessed whether: (a) there were differences in the retrieval of object-color knowledge from verbal and visual modalities; and (b) retrieval modality influenced memory as indexed by priming.

The available evidence suggests that retrieval of object-color knowledge may be modality-specific. First, neuropsychological evidence suggests a distinction between visual and verbal object-color information, for instance in knowing that a banana is yellow without consulting a visual representation (Beauvois, 1982; Beauvois and Saillant, 1985; Tanaka et al., 2001). Related to this, in studies of the development of object-color knowledge in children, younger children appear to store most of this knowledge via verbal rather than visual processing. For instance, when presented with pictures of a yellow and red banana a young child may not choose correctly, however when asked “What color are bananas?” is able to answer that bananas are yellow (Davidoff and Mitchell, 1993; Gleason et al., 2004). Note as well that, (a) visual object-color information can be accessed from a shape representation (Price and Humphreys, 1989) or via a verbal object-color representation through the use of mental imagery (Davidoff, 1991; Tanaka et al., 2001) and (b) verbal object-color information may have direct access to object and color names (Beauvois, 1982; Beauvois and Saillant, 1985; Davidoff and Mitchell, 1993; although see Tanaka et al., 2001). Second, two studies highlight differences in knowledge retrieval from verbal and visual modalities, and although the paradigms are very different from each other and from the present task they are suggestive. Naor-Raz et al. (2003) used a variation of the (Stroop, 1935) paradigm whereby participants named the colors of diagnostically colored objects (where color is a cue to identity, as in strawberry; Tanaka and Presnell, 1999) or object names. For objects, a Stroop-like effect was evident with slower naming times for atypical (e.g., blue apple) than typical stimuli. In contrast, for object names this pattern was reversed and there were faster naming times for atypical stimuli. They also found that object names, but not objects, facilitated subsequent lexical decisions to associated concepts (for instance, apple primed pie when deciding whether the stimulus was a word or nonword). Naor-Raz et al. (2003) suggested that object names had more ready access to object-color information than visual objects in the color naming task. More recently, Huettig and Altmann (2010) presented the names of diagnostically colored objects within an auditory contextual sentence whilst monitoring eye movements (e.g., “The man thought about it for a while and then he looked at the frog and decided to release it back into the wild”). Object names, but not black-and-white photographs or line drawings, provided access to stored object-color information which in turn shifted overt attention to objects in the display with the same surface color. In some contexts then, it appears that object names can have more effective access to object-color information than visual objects.

In the present study, we assessed priming from object naming onto a lexical-semantic matching task. The rationale was (a) to use object naming to tap the object-color knowledge system whereby links between object and color representations may be activated at a visual (Price and Humphreys, 1989) semantic (Davidoff, 1991; Davidoff et al., 1997; Tanaka et al., 2001) or lexical level (Naor-Raz et al., 2003). Supporting this notion, there is considerable evidence that naming a familiar object is normally mediated at least by three kinds of pre-existing representation: visual input is matched to a stored visual representation of object shape; accessing this stored shape representation enables further access to a semantic representation which provides the basis for recognition; and in order to name a visually presented object a number of additional post-semantic lexical stages involved in name selection and production have also been proposed (Indefrey and Levelt, 2004). Models differ as to whether during naming information transmission at some prior stage stops or is completed before processing at a subsequent stage begins (Schriefers et al., 1990; Levelt et al., 1999) or whether it is continuously fed forward and backward between either some or all representational stages (Humphreys et al., 1995; Rapp and Goldrick, 2000). Nevertheless, in a long-term priming paradigm as used here one would expect activation from initial naming to spread to all parts of the object-color system (e.g., Lloyd-Jones and Humphreys, 1997a,b). In addition, we proposed that (b) long-term priming arises from the activation of processing components engaged across study and test tasks (for a recent review, see Cabeza and Moscovitch, 2013) and therefore from a subset of components activated during object naming and the lexical-semantic matching task. In lexical-semantic matching participants encountered a visually presented object name (Experiment 1) or object shape (Experiment 2) paired with either a color patch or color name. The pairings could either match whereby both were consistent with a familiar object (e.g., *strawberry* and *red*) or mismatch (*strawberry* and *blue*). Accordingly, we proposed that successful responses on match trials required access to lexical-semantic information about familiar objects and their prototypical colors. We used the matching task to probe knowledge about familiar objects and their colors pre-activated during object naming.

In Experiment 1 we assessed the retrieval of object-color information from object names. We examined the priming of (a) *object name+color patch* (same object name and physical color as at study); (b) *object name+color name* (same object name and color name, where the color name corresponded to the object color at study); and (c) object *name alone* (same object name as at study but with a different color patch or color name to that encountered at study); as compared with (d) *control* (an object name and color patch or color name that had not been encountered previously). The logic was straightforward: if there was similar priming for the conditions where the same object name+color was processed across study and test as compared with the conditions where only the same name was processed across study and test, then the object-color associations activated during the naming task were not utilized by the system(s) mediating performance. In contrast, if there was greater priming for the conditions where the same object name+color was processed across study and test as compared with the conditions where only the name was processed across study and test, then the object-color associations activated during the naming task were utilized by the system(s) mediating performance (for the same logic, see Lloyd-Jones and Nakabayashi, 2009; Lloyd-Jones et al., 2012; and others). We predicted that object-color information would contribute to priming in the lexical-semantic decision task and so there would be greater priming for object name+color as compared with the name alone. We also predicted that priming would be modulated by the nature of the color retrieval cue. There is flexibility, according to processing demands, in the encoding and/or retrieval operations of the memory system for certain object properties (for a review, see Roediger and Srinivas, 1993). For instance, if the task requires a judgment about object size then size may influence priming but otherwise it may not do so (Srinivas, 1996). Similarly, object color can influence priming when it is made relevant to the task but under other circumstances it may not do so (Vernon and Lloyd-Jones, 2003). Complementary evidence also comes from a short-term priming paradigm used by Yee et al. (2012) who found color-name priming (e.g., the word *emerald* primed *cucumber*) but only when attention had been drawn to the color feature by participants previously completing a color-word Stroop task. Our main focus here was the color patch condition where the aim was to probe visual object-color memory. We proposed that the processing component engaged by the physical color during both encoding (as part of the visual object) and retrieval (by the color patch) was visual object-color information. Consistent with this idea, Price and Humphreys (1989) have shown that surface color information contributes at a visual level to object categorization and naming although we note that they found color effects only when color covered the surface of the object (not when it was used as a background color) and here color was only partially superimposed on the name (or shape in Experiment 2). Nevertheless, if we are correct, the pre-activation of visual object-color information by object naming should mediate object name+color processing in the lexical-semantic matching task and produce priming. In contrast, we expected that the color-name cue would not encourage the retrieval of visual object-color memory as effectively because it would have to do so via the retrieval of verbal object-color information and a process of visual imagery. Rather, we proposed that the color-name cue predominantly would encourage the retrieval of verbal object-color information and as a consequence we would observe reduced long-term priming as compared with the color patch condition.

Finally, there was the intriguing possibility that interference might arise for the color-name cue because of the frequent repetition of a relatively small set of visually presented color names during retrieval. Repetition priming can have short-term negative consequences whereby retrieving a word can interfere with retrieving subsequent words from the same semantic category (for reviews, see Abdel Rahman and Melinger, 2009; Oppenheim et al., 2010). In *semantic blocking* for instance, objects are named more slowly in the context of items from the same category as compared with items from various semantic categories (Belke et al., 1985; Kroll and Stewart, 1994). These semantic context effects on language production are generally short-lived, although interference can arise across filler trials (Wheeldon and Monsell, 1994; Damian and Als, 2005; Howard et al., 2006) and in one study across experimental blocks (Vitkovitch and Humphreys, 1991). Persisting negative effects have been proposed to arise from a combination of (a) *shared semantic activation*, so that activation of one particular word or picture activates both itself and semantically-related concepts; (b) *priming*, whereby the activation/retrieval of a lexical-phonological representation facilitates the subsequent activation/retrieval of that representation, through item-specific mappings from semantics to lexical-phonology; and (c) *competition*, so that item-specific mappings from semantic to lexical-phonological representations also result in the activation of a number of lexical competitors (Howard et al., 2006; Oppenheim et al., 2010). Competition may be resolved either by lateral inhibition within the lexicon (Howard et al., 2006; but see Navarrete et al., 2010) or learning, namely small and persistent experience-driven adjustments to the mappings between semantic and lexical representations which involve strengthening the mappings for the word that is produced and at the same time weakening the mappings for semantically-related words (Oppenheim et al., 2010). Oppenheim et al. (2010) suggest that these negative effects will arise in other tasks which involve semantic-based lexical-phonological processing. Now, when color names are presented with object names here, the three conditions described previously are satisfied. On the basis of neuropsychological and developmental evidence (Beauvois, 1982; Beauvois and Saillant, 1985; Mitchell and Davidoff, 1993) there are direct links between verbal object-color (a form of semantic knowledge) and lexical-phonological color-name representations. In addition, during word recognition and reading aloud, lexical-phonological representations are always activated (although not necessarily fully specified; Frost, 1998; Coltheart et al., 2001). So, on this basis there is (a) shared semantic activation, whereby color names provide access to verbal object-color knowledge in order to make a semantic decision; (b) short-term priming, a small set of visually presented color names are presented repeatedly and item-specific priming may arise from the mappings between verbal object-color and lexical-phonological color-name representations; and (c) lexical competition, item-specific mappings from verbal object-color knowledge to lexical-phonological representations may result in the activation of a number of lexical-phonological competitors. In a similar fashion, competition may also arise at the level of lexical-orthographic representations as there is evidence that when a word is visually presented there is activation of its orthographic/phonological competitors (McCann and Besner, 1987; Andrews, 1992). In sum, it is plausible that semantic interference will produce longer response times for color names as compared with color patches.

Furthermore, concerning long-term priming, it follows that in a system where information is transmitted continuously between representational stages, activation of visual object-color knowledge from prior object naming may exaggerate competition between subsequent verbal object-color representations and as a consequence inhibit the retrieval of color-name representations relative to the control condition. Consistent with this notion: (a) in an analogous fashion, studies have shown that visual object similarity based on shared shape features can have repercussive effects, exaggerating competition at subsequent semantic and lexical stages of the object naming system (e.g., Vitkovitch et al., 1993; Humphreys et al., 1995; Lloyd-Jones and Humphreys, 1997a,b) and (b) as Damian and Als (2005) describe, a number of studies have demonstrated that the retrieval of an object name can result in that item being a more powerful competitor on subsequent trials in which items from the same category are named (Vitkovitch and Humphreys, 1991; Wheeldon and Monsell, 1994; Vitkovitch et al., 2001). For instance, using a naming to deadline procedure where participants have to respond before they are ready resulting in various kinds of error, Vitkovitch and Humphreys (1991) found that such errors were often *perseverative*—the names of category members which were targets during an earlier block of trials. In sum, we expect inhibitory priming from the color-name retrieval cue.

## Materials and methods

### Participants

There were 189 participants in all; 21 took part in a preliminary color agreement study, 84 took part in Experiment 1 and 84 in Experiment 2. All were undergraduates at the University of Kent and participated for course credit. All had normal color vision and normal or corrected-to-normal visual acuity.

### Stimuli and apparatus

The initial pool of stimuli were color photographs of 75 common objects from number of different categories. Most pictures were taken from an internet website (www.PhotoObjects.net) with a subset selected via an internet image search using the Google search engine. The objects were selected on the basis that each object had a single diagnostic color and where possible the surface color of each object was based on color agreement scores obtained by Joseph (1997) and Vernon and Lloyd-Jones (2003). We used the imaging software Adobe Photoshop CS2 to create 3 versions of each object: a correctly colored object, a grayscale object, and an incorrectly colored object. To convert correctly colored objects to grayscale all the images were converted into the Lab color mode allowing the separation of luminosity (i.e., the lightness component that can range from 0 to 100) from the color. The lightness channel was then converted into the grayscale channel by using the grayscale mode. To convert correctly colored objects to incorrectly colored objects, we rotated the correct colors across objects ensuring that correctly and incorrectly colored objects were matched for color frequency and luminosity, with the constraint that each incorrectly colored object was not similar to the correctly colored version (e.g., we did not replace the green of a lettuce with the green of a cucumber). The incorrectly colored objects were created by selecting the surface color of an object which was pasted onto another object by using the color replacement tool. The brightness of the color-replaced object was adjusted by using the brightness contrast tool. The luminosity of grayscale images was also closely matched to that of the colored objects (i.e., there were differences only in the range of 10–15 in the Adobe lightness component).

We then examined color agreement between the surface color of each object (i.e., the color that was assigned to each object by the experimenter) and participants' knowledge of the prototypical color of each object. In a self-paced task, 21 participants wrote down the color of each object in both a perception and a memory condition. In the perception condition, each of 75 correctly colored objects was shown, in random order, one at a time on the computer screen until a response was made, and participants wrote down what they considered to be the surface color of the object. In the memory condition, participants were given the list of the names of 75 objects, and were asked to assign to each object what they thought was the object's most prototypical color from memory. The order of conditions was counterbalanced across participants. It was clear that 15 objects had strong perception-memory color disagreement (i.e., apple, aubergine, chick, chicken. elephant, giraffe, grapes, lion, onion, peach, pepper, pineapple, tank, tulip, and turtle). The surface color of 11 of these objects were then re-colored into the color which the participants thought was the most prototypical color (the surface color of 3 objects remained unchanged because participants had reported the internal rather than external color and a fourth object was excluded because name agreement was low). Finally, we selected 60 objects with the highest perception-memory agreement and prepared them for the lexical-semantic matching task: average color agreement was 80%.

**Figure 1:**
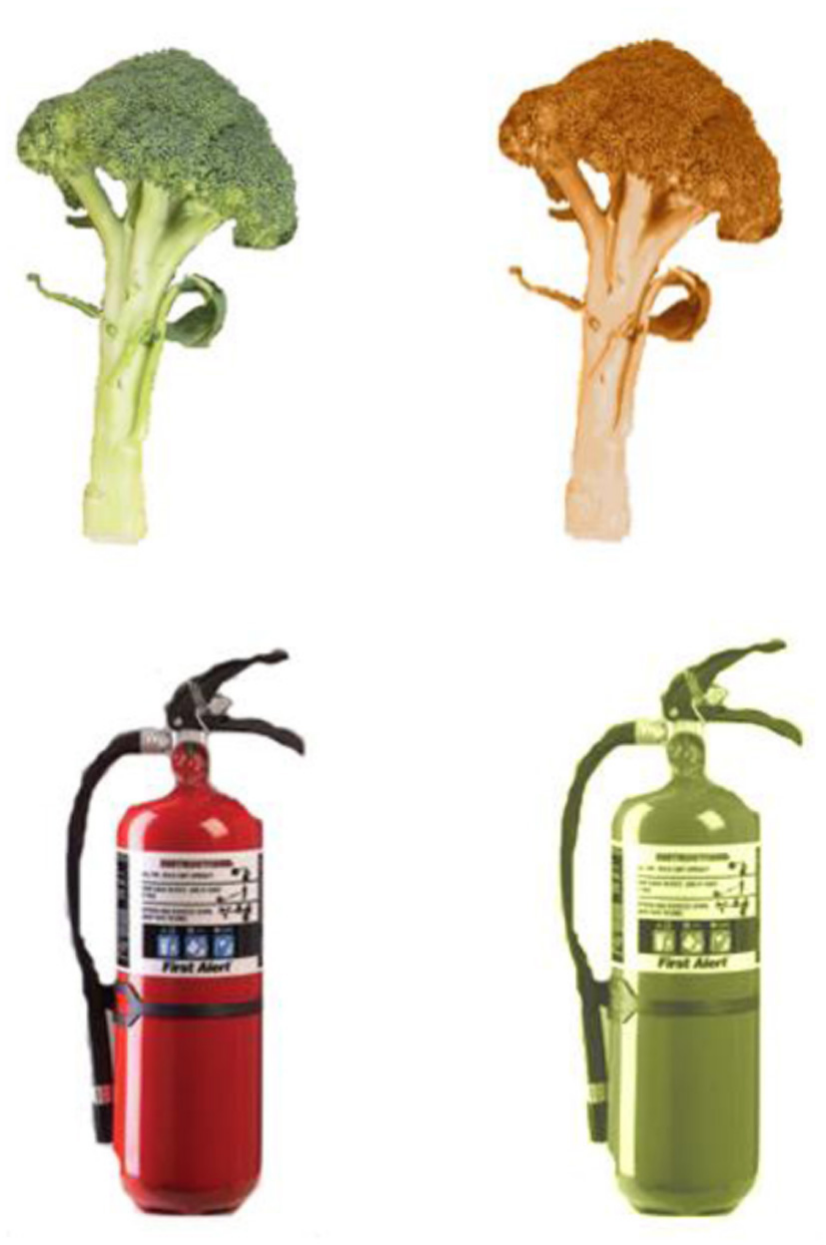
**Examples of correctly and incorrectly colored objects presented in the object naming task**.

**Figure 2:**
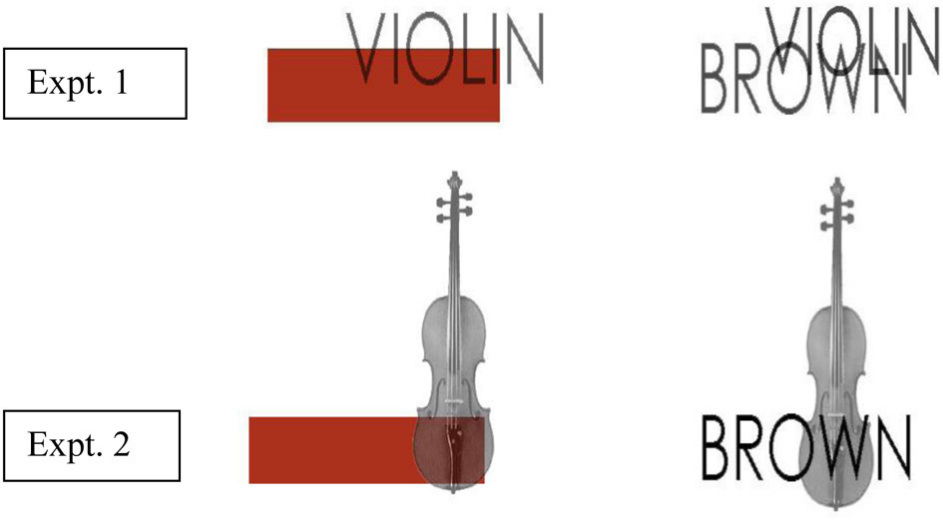
**Examples of object and color format conditions in the lexical-semantic matching task for *violin***. The test conditions were: Experiment 1; object name + color patch; object name + color name. Experiment 2; object shape + color patch; object shape + color name.

For the lexical-semantic matching task we created color patches using the same correct and incorrect colors used to color the surface of the object in the preliminary study described above and in the object naming task used at study in Experiments 1 and 2. We selected the surface color of the object and pasted that color onto a box using the color replacement tool. Each color patch was partially superimposed onto either the object name or a grayscale image of the object with the color patches positioned equally to the top left, top right, bottom right and bottom left of the object or word. We did this to control as far as possible for potential differences in attention across the conditions. Participants' attention here was to a single object with name/shape+color conjoined. In contrast, had name/shape vs. color been presented spatially separately this might have encouraged subjects to attend more to either the name/shape or the color and possibly to do so to a different extent in the various conditions. The average size of the color patches was matched with that of the color words. These sizes were also equivalent to the size of the objects and object names which were also matched with each other: 4 cm (h) × 6 cm (w). This was achieved by pairwise matching the size of each object with the size of the corresponding object name and also pairwise matching this size with the size of the corresponding color patch and color name. The font was Century Gothic in upper case 27 point. For the object-name/color-name condition we were concerned that the two components to the stimulus may not be as perceptually discriminable as the components in the other conditions and we therefore adjusted the opacity of the object and color words to 70% in order to make them more readable. A list of the stimuli are given in Supplementary material. Figure 1 provides examples of correctly and incorrectly colored objects presented in the object naming task and Figure 2 provides an example of each object and color format combination used in the lexical-semantic matching task for *violin*. The experiment was conducted using SuperLab Pro (Version 2.0.4) on a PC, with a microphone via a voice key system (Cedrus SV-1).

## Experiment 1

### Design

The experiment comprised two phases: (a) a study phase where correctly and incorrectly colored objects were named followed by (b) a test phase in which a lexical-semantic matching task was performed. In the lexical-semantic matching task participants encountered a visually presented object name paired with either a color patch or color name. The pairings could either match whereby both were consistent with a familiar object (e.g., *strawberry* and *red*) or mismatch (e.g., *strawberry* and *blue*). Accordingly, we proposed that in this experiment (but not Experiment 2) successful responses on match trials required access to lexical-semantic information about objects and their color properties. Therefore, we were most interested in the effects of *priming from naming onto match trials corresponding to correctly colored objects at test* in the following conditions: (a) *object name+color patch* (same object name and physical color as at study); (b) *object name+color name* (same object name with a color name corresponding to the object color encountered at study); and (c) object *name alone* (same object name as at study but with a different color patch or color name from that at study); as compared with (d) *control* (an object name and correct color patch or color name that had not been encountered previously).

To provide a fully balanced design there were 20 objects in the study phase with correct color and 20 with incorrect color. In the test phase there were 60 stimuli, with half in the correct color and half in an incorrect color. Within each of these two conditions at test, stimuli could be the same as at study (i.e., same correct color or same incorrect color; 20 stimuli in all), they could have changed from study (i.e., changed from correct to incorrect color or vice versa; 20 stimuli in all) or they were new stimuli (correct and incorrectly colored; 20 stimuli in all) which provided baselines against which to compare the effects of priming, where the comparison was always within correctly or incorrectly colored conditions at test. In this way, 6 lists of 10 test items were rotated through the study and test conditions, to ensure that each stimulus appeared equally often in each of the conditions across the experiment.

### Procedure

The study task was to name the object, out loud, as quickly and accurately as possible. In the test phase participants were required to make speeded key press responses to indicate whether or not the color was typical of the object. Half the participants pressed the A key for *match* responses (that the color was typical of the object) and the L key for *mismatch* responses. For the other half of participants the key mapping was reversed. Participants each received 3 practice trials. The test phase followed on from the study phase after a few minutes which was also used to brief participants as to the nature of the task: study and test phases together took approximately 15–20 min to complete. For both study and test phases each trial began with a fixation cross for 250 ms, followed by a 500 ms blank screen, and then by the stimulus which remained on screen until a response had been made.

### Results and discussion

For both this experiment and Experiment 2 we adopted the following approach. First, to ascertain whether there was a positive effect of correct color on object naming at study we directly compared correctly and incorrectly colored objects. This was important because if there was no effect of color at study then we would not expect to obtain color priming at test because color knowledge had not been contacted during the course of object naming. For priming however, we were interested only in the data for *match trials corresponding to correctly colored objects at test* because the focus of this experiment was on the retrieval of pre-existing lexical-semantic knowledge concerning real objects and their colors rather than novel representations constructed on-line during learning which is the case for mismatch trials corresponding to incorrectly colored objects at test (Musen et al., 1999; Vernon and Lloyd-Jones, 2003). Indeed, we would not expect pre-existing long-term links in semantic or lexical memory between names and colors for incorrectly colored objects (Davidoff, 1991). We therefore report the findings for mismatch trials corresponding to the priming of incorrectly colored objects at test in Supplementary material. As we expected, priming of incorrectly colored objects arose for name information alone in Experiment 1 and for shape information alone in Experiment 2: there was no priming of either name or shape in combination with color. Note also, if we analyse the response times for correctly and incorrectly colored objects at test together there is a 3-way interaction, *F*_(2, 144)_ = 6.26, *p* = 0.002, demonstrating that the findings presented here are robust.

For the naming task at study, a trial was scored an error if: (a) participants provided an incorrect response according to the list of names in Supplementary material. Note, the average name agreement for the objects used in the study was 89%. This approach is more stringent than accepting alternative names produced by some proportion of participants nevertheless it is clear that the study had sufficient power (see summary statistics); (b) the naming latency was 2.5 standard deviations above or below the mean for that participant; or (c) a machine error occurred. In addition, responses to test trials where an error had been made to the object on the corresponding study trial were not excluded. If they were excluded it may have resulted in the removal of objects with names that were intrinsically more difficult to produce and since data from such objects would be excluded from the primed but not the unprimed conditions, this might have resulted in an illusory priming effect. Including such data is a conservative procedure (Wheeldon and Monsell, 1992). We report effect sizes, estimated using partial eta-squared (η*p*^2^) which according to generally accepted criteria ranged from medium to large (Cohen, 1988; 0.01 = small, 0.06 = medium, 0.14 = large). For a summary of the data see Table 1.

**Table 1:** **Experiment 1: Mean response times, standard error (SE), and percentage correct (%) for object name and color in the lexical/semantic matching task**.

	**Object name + color**			**Name**			**Control**		
	**Mean**	***SE***	**%**	**Mean**	***SE***	**%**	**Mean**	***SE***	**%**
**Format**									
Patch	882	34.2	84.3	922	36.3	81.2	933	41.4	79.6
Name	1160	38.2	95.1	1110	44.6	92.9	1103	43.9	90.3

#### Study (object naming)

For the analysis of variance (ANOVA), the within-subjects factor was *color* (correct vs. incorrect). For response times, there was a main effect of *color*, with shorter response times for correctly colored objects (917 vs. 993 ms, respectively), *F*_(1, 82)_ = 31.61, *p* = 0.000, η*p*^2^ = 0.29. For accuracy, there was also a main effect of *color*, with greater accuracy for correctly colored objects (87 vs. 75%), *F*_(1, 82)_ = 35.10, *p* = 0.000, η*p*^2^ = 0.30. In sum, correct color benefited object naming performance.

#### Test (lexical-semantic decision)

For the ANOVA, the within-subjects factor was *priming* of (a) object name+color (same object name and color as at study) and (b) object name alone (same object name as at study but presented with a different color) compared with (c) control (a correct object name and color that had not been encountered previously). The between-subjects factor was *color format* (color patch vs. color name). We also included the variable *stimulus list* from the rotation design (here and in Experiment 2) in order to increase power as recommended by Pollatsek and Well (1995), nevertheless if we exclude this factor the findings remain unchanged (note also, because of the counterbalanced design across both study and test, no item analyses are reported; Raaijmakers et al., 1999).

For response times, there was a main effect of *color format*, *F*_(1, 72)_ = 15.58, *p* = 0.000, η*p*^2^ = 0.18, with longer response times to color names as compared with color patches. There was also a *priming* x *color format* interaction *F*_(2, 144)_ = 5.55, *p* = 0.005, η*p*^2^ = 0.07. Planned comparisons (*t*-tests) revealed facilitatory priming for object name+color as compared with the control condition in the color-patch condition, *p* < 0.005. There was no priming for name alone compared to control. Note also, response times were shorter for object name+color as compared with the name alone condition, *p* < 0.05. For the color-name condition, there was inhibitory priming for object name+color as compared with the control condition, *p* < 0.005. There was no priming for name alone condition compared to control. Note also, response times were longer for object name+color as compared with name alone, *p* < 0.005.

For accuracy, there was a main effect of color format, *F*_(1, 72)_ = 49.58, *p* = 0.000, η*p*^2^ = 0.41, with less accuracy for color patches as compared with color names. There was also a main effect of *priming*, *F*_(2, 144)_ = 3.80, *p* = 0.025, η*p*^2^ = 0.05, with greater accuracy for object name+color as compared with the control condition, *p* < 0.005. The color format × priming interaction was not significant, *F*_(2, 144)_ = 0.05, η*p*^2^ = 0.01, *p* = ns.

Together, these findings indicate that long-term mappings between object names and prototypical colors were activated in memory. However, depending on the nature of the color cue, prior activation either helped or hindered memory retrieval. When the cue was a color patch, memory retrieval was enhanced and when the cue was a color name, memory retrieval was inhibited. There was some evidence that participants traded speed for accuracy and this contributed to the overall effect of color format, with longer response times but also greater accuracy in the color name condition. However, this cannot account for the contrasting influence of the color retrieval cue on priming. Indeed, longer baselines are normally associated with an increase in facilitative priming rather than inhibition which was observed here (Ostergaard, 1994). Moreover, there was no significant correlation between response time and accuracy for any condition: Pearson's *r* values ranged from −0.19 to 0.18. Rather, in combination with the effects of priming we suggest that longer lexical-semantic decision times overall for color names were driven predominantly by semantic interference.

## Experiment 2

We have argued that important differences in knowledge activation can arise according to the retrieval process. In particular, object names can have more effective access to object-color information than visual objects. To test this account further, we examined whether the findings from Experiment 1 with object names would be reproduced when object shapes provided access to object-color information. Here, we predicted that object shape, but not color, would be used by the memorial system mediating performance and so there would be equivalent priming for object shape+color as compared with the shape-alone condition. The design and procedure was the same as Experiment 1 with the exception that in the test phase decisions were made to matching or mismatching grayscale objects paired with color patches or color names. For a summary of the data see Table 2.

**Table 2:** **Experiment 2: Mean response times, standard error (SE), and percentage correct (%) for object shape and color in the lexical/semantic matching task**.

	**Object shape + color**			**Shape**			**Control**		
	**Mean**	**SE**	**%**	**Mean**	**SE**	**%**	**Mean**	**SE**	**%**
**Format**									
Patch	1006	32.1	98.6	1020	43.3	98.7	1083	40.5	98.4
Name	846	25.1	98.8	864	29.4	99.1	936	27.1	98.9

### Results and discussion

#### Study (object naming)

For response times, there was a main effect of *color* with shorter response times to correctly colored objects (946 vs. 1020 ms, respectively), *F*_(1, 82)_ = 44.70, *p* = 0.000, η*p*^2^ = 0.35. For accuracy, there was also a main effect of *color* with greater accuracy for correctly colored objects (86 vs. 77%), *F*_(1, 82)_ = 13.98, *p* = 0.000, η*p*^2^ = 0.96. In sum, correct color benefited object naming performance.

#### Test (lexical-semantic decision)

For response times there was a main effect of *priming*, *F*_(2, 144)_ = 18.13, *p* = 0.000, η*p*^2^ = 0.21. Planned comparisons revealed facilitatory priming for object shape+color as compared with the control condition, *p* < 0.001, and also for shape alone as compared with the control condition, *p* < 0.001. (There was no difference between object shape+color and shape alone, *p* = 0.275.) There was also a main effect of *color format*, *F*_(1, 72)_ = 11.62, *p* = 0.001, η*p*^2^ = 0.14, with longer response times to color patches as compared with color names. For accuracy, there was a main effect of *color format*, *F*_(1, 72)_ = 4.5, *p* = 0.037, η*p*^2^ = 0.06, with less accuracy for color patches as compared with color names. The color format x priming interaction was not significant, *F*_(2, 144)_ = 0.22, η*p*^2^ = 0.01, *p* = ns.

The main finding was that object shape, but not color, was used by the memorial system mediating performance. This is consistent with object shape providing a less effective retrieval cue for object-color knowledge than the object name in the lexical-semantic retrieval task. Moreover, if we directly compare priming across Experiments 1 and 2, there is a three-way interaction, *F*_(2, 328)_ = 3.84, *p* = 0.022, η*p*^2^ = 0.02, which provides strong evidence for the claim that names are better at activating color knowledge than shapes. Note, we are not suggesting that object-color knowledge cannot be used at a visual level of analysis, as there is convincing evidence for mappings between object shape and visual object-color information (e.g., Price and Humphreys, 1989; Bramão et al., 2012; Lloyd-Jones et al., 2012). Rather, we are proposing that object shape does not provide an effective retrieval cue in the lexical-semantic matching task.

Independently, there was also an effect of color format with poorer performance for color patches (longer response times and less accuracy) as compared with color names. This contrasts with Experiment 1, where we observed longer response times for color names. Our account of semantic interference requires shared item-specific semantic activation whereby verbal object-color knowledge is activated for a particular object and also other semantically-related objects (which in turn produces lexical-phonological competition). Now, in Experiment 1 this was evident because presentation of the object name and color name specified a verbal object-color entry. Here however, we propose that the object shape initially contacted visual object-color information because object shape and visual object-color information are tightly interconnected (Price and Humphreys, 1989) and this was sufficient to make a decision. This meant that one of the conditions necessary for semantic interference was not met. Poorer performance for color patches likely reflected the fact that they share visual similarity (e.g., orange-red, blue-green) whereas color names do not, and this resulted in the activation of a greater number of visual object-color alternatives which increased competition at the visual level when making a decision. Supporting this notion, words corresponding to stimuli from the same semantic category are no more physically similar than words corresponding to stimuli from different semantic categories (Carr et al., 1982) whereas pictures corresponding to stimuli from the same semantic category can share physical resemblance; for instance, animals, fruit and vegetables (for a recent review, see Lloyd-Jones and Nettlemill, 2007). For physical colors, as Braisby and Dockerell (1999) have described, particular instances can fall under different color terms. For instance, a color patch may be equally considered an instance of orange or red, just as dictionaries define olive to be yellow-green, aquamarine to be greenish-blue, and burgundy to be blackish-purple to purplish-red (Collins English Dictionary, 2014). So, for color patches their visual similarity influenced performance when the task was performed on the basis of visual information.

## General discussion

The majority of previous work on object-color knowledge has focused on object recognition and found moderate effects of color on categorization and naming (for a recent review and meta-analysis, see Bramão et al., 2011). Here, we examined the retrieval of object-color knowledge from long-term memory. We developed a novel paradigm, which we argue selectively tapped the retrieval of prototypical colors of familiar objects from object names, and used it to examine long-term priming from object naming onto lexical-semantic decisions about objects and their colors and the use of modality-specific access procedures for the retrieval of stored object-color knowledge. We found that priming varied with the nature of the retrieval process. Object-color priming arose for object names (Experiment 1) but not object shapes (Experiment 2) and beneficial effects of priming were observed for color patches whereas inhibitory priming arose with color names. The findings have implications for understanding how object knowledge is retrieved from memory and modified by learning.

The observation that object names enabled the long-term retrieval of object-color information stored in memory complements work on language comprehension showing that visual and motor representations of objects can be activated during word and sentence processing (for a review, see Zwaan, 2004; although see also Rommers et al., 2013). Such findings have often been interpreted in terms of sensorimotor theories of semantic memory whereby object knowledge is represented in a modality-specific rather than amodal fashion (Barsalou, 1999; although see Mahon and Caramazza, 2008). Moreover, our findings support (a) the claim that object names can be more effective than object shapes in retrieving stored object-color knowledge (Naor-Raz et al., 2003); and (b) the independence of object color from shape knowledge (Miceli et al., 2001). The fact that object names were particularly effective object-color retrieval cues also complements recent work by Lupyan and Thompson-Schill (2012) showing that, across short delays in picture verification tasks, semantic information is activated more effectively through the use of verbal labels (such as *cat*) as compared with non-verbal cues (such as the sound of a cat meowing) or words that do not directly refer to the object (the word *meowing*). They suggest that object names are particularly effective because they specify the concept precisely whereas other memory cues may activate a more idiosyncratic semantic representation. Here, object names shared little physical similarity across exemplars and so activated few semantic object-color alternatives. In contrast, object shapes were visually similar (for instance, exemplars came from fruit, vegetable and animal categories) and in a system where information is continuously fed forward the co-activation of a number of competing visual representations will activate a greater number of semantic object-color alternatives (Vitkovitch et al., 1993; Lloyd-Jones and Nettlemill, 2007). So, for object shapes it is likely that access to stored object-color knowledge was more variable.

We also observed both facilitatory and inhibitory priming which was modulated by the color retrieval cue in the lexical-semantic matching task. As we shall describe, both forms of priming can be explained by learning within a lexical-semantic system comprising visual and verbal object-color knowledge and object and color names. Long-term repetition priming normally has a beneficial effect on performance and is contingent upon the overlap of perceptual, semantic, lexical and response-related processes engaged during encoding and retrieval so that priming is reduced when an item is presented in a different modality or format from study to test (Durso and Johnson, 1979; Rajaram and Roediger, 1993). In addition however, activating/retrieving a particular lexical item can have an adverse short-term effect on the retrieval of other semantically-related lexical items (Howard et al., 2006; Abdel Rahman and Melinger, 2009; Oppenheim et al., 2010). Here, we argue that when the physical color of the object was present during both encoding (as part of the object that was named) and retrieval (an object name+color patch was the memory cue) pre-existing mappings between object names and visual object-color knowledge were activated and mediated facilitatory priming. In contrast, when retrieval was cued by color names two modality-specific conditions arose which together were likely to encourage semantic interference: (a) there was less overlap in processing relative to the color patch condition because the physical color was encoded but color names were presented at retrieval. This meant that the potential benefit of long-term priming was reduced relative to the color patch condition and this allowed any effects of interference to become more apparent; and (b) color names, but not color patches, map directly onto verbal object-color knowledge (Beauvois, 1982; Beauvois and Saillant, 1985; Davidoff and de Bleser, 1993; Davidoff and Mitchell, 1993). We suggest that repeated access to verbal object-color knowledge from color names accrued categorical activation in the verbal object-color system which in turn increased competition between color names at the level of phonology and/or orthography. Long-term inhibitory priming was observed because prior object naming exaggerated effects of semantic interference by making those items particularly powerful competitors in the verbal object-color system (cf., Vitkovitch and Humphreys, 1991; Damian and Als, 2005).

Finally, in previous work we have discussed whether effects of color on object-based memory retrieval reflect either established long-term mappings between object shape and color knowledge or the creation of new temporary short-term perceptual bindings between shape and color (Vernon and Lloyd-Jones, 2003; Lloyd-Jones and Nakabayashi, 2009). For instance, in an event-related potential study Lloyd-Jones et al. (2012) observed color priming for objects in a colored-object decision task (“Is this object correctly colored?”) from prior object naming. Priming was equivalent for correctly and incorrectly colored objects and evident early in the time course of processing (around 200 ms after stimulus onset). They suggested that the effects arose from perceptual learning which can take place after just a single study trial and has been observed for novel objects (Graf and Schacter, 1989; Wang and Bingo, 2010). Their findings contrast nicely with those presented here where we observed effects of color on memory for familiar but not novel combinations of names and colors. It is likely therefore, that color can influence memory retrieval in a number of ways. We have developed a new paradigm which combined with priming selectively engages the long-term mappings between object names and object-color knowledge and so provides a powerful tool for studying long-term object representation and retrieval.

### Conflict of interest statement

The authors declare that the research was conducted in the absence of any commercial or financial relationships that could be construed as a potential conflict of interest.
