# Supplementary material for: Long-term repetition priming and semantic interference in a lexical-semantic matching task: tapping the links between object names and colors
Source: Front Psychol. 2014 Jun 24;5:644. doi: 10.3389/fpsyg.2014.00644 (PMC4068293; doi:10.3389/fpsyg.2014.00644)
Supplement: Supplementary file 1 [file Presentation1.PDF]

Appendix 1

Full list of stimuli

Apple, Aubergine, Buffalo, Banana, Broccoli, Carrot, Caterpillar, Cauliflower, Cherry, Chick, Chicken, Corn, Cucumber, Deer, Desk, Dog, Duck, Elephant, Fire Engine, Fire Extinguisher, Flamingo, Fly, Frog, Giraffe, Grapes, Grasshopper, Guitar, Horse, Ladybird, Lemon, Lettuce, Lion, Lizard, Lobster, Monkey, Onion, Orange, Peach, Peanut, Pear, Peas, Pepper, Pig, Pineapple, Polar Bear, Potato, Pumpkin, Raspberry, Rose, Snail, Stop sign, Strawberry, Sunflower, Tank, Tiger, Tomato, Turtle, Violin, Wardrobe, Watermelon.

Appendix 2

Experiment 1: Incorrectly coloured objects at test (lexical-semantic decision)

For response times, there was a main effect of *priming*, F(2,144)=4.91, p=.009, ηp^2=.06. Planned comparisons (t-tests) revealed facilitatory priming for object name+colour (985ms, *SE*=26.15) as compared with the control condition (1022ms, *SE*=29.23), p<.01, and also for name alone (983ms, *SE*=27.15) as compared with the control condition, p<.001. There was also a main effect of *colour format*, F(1,72)=11.82, p=.001, ηp^2=.14, with longer response times to colour names (1087ms, *SE*=37.21) as compared with colour patches (906ms, *SE*=37.21). For accuracy, there was a *priming x colour format* interaction, F(2,144)=8.54, p=.000, ηp^2=.10. For colour patches, there was greater accuracy for control (95.5%, *SE*=4.45) compared with object name+colour (89.3%, *SE*=1.51) p<.01, and also control compared with name alone (91.3%, *SE*=1.35) p<.05. For colour names, there was greater accuracy for object name+colour (92.1%, *SE*=1.49) compared with control (86.4%, SE=1.56) p<.05, and a marginally non-significant difference between name alone (90.9, *SE*=1.33) and control, p=.073.

These findings indicate that for incorrectly coloured objects, name information alone was retrieved from memory. Response times were also longer for colour names than colour patches.

Experiment 2: Incorrectly coloured objects at test (lexical-semantic decision)

For response times, there was a main effect of *priming*, F(2,144)=7.05, p=.001, ηp^2=.08. Planned comparisons revealed facilitatory priming for shape alone (961ms, *SE*=22.71) compared with control (1012.81ms, *SE*=24.48) p<.001. There was no difference between shape+colour (984ms, *SE*=23.71) and control, p=.130. For accuracy, there was a main effect of *priming*, F(2,144)=6.49, p=.002, ηp^2=.08. Planned comparisons revealed greater accuracy for shape alone (92.86, *SE*=.87) compared with control (89.17, *SE*=1.31) p<.05. There was no difference between shape+colour (87.38, *SE*=1.35) and control, p=.186.

These findings indicate that for incorrectly coloured objects, shape alone was retrieved from memory.
